# The fruit fly *Drosophila melanogaster* as a screening model for antiseizure medications

**DOI:** 10.3389/fphar.2024.1489888

**Published:** 2024-12-10

**Authors:** Florian P. Fischer, Robin A. Karge, Henner Koch, Aaron Voigt, Yvonne G. Weber, Stefan Wolking

**Affiliations:** ^1^ Department of Epileptology and Neurology, RWTH Aachen University, Aachen, Germany; ^2^ Department of Neurology, RWTH Aachen University, Aachen, Germany; ^3^ JARA-BRAIN Institute Molecular Neuroscience and Neuroimaging, Forschungszentrum Jülich GmbH and RWTH Aachen University, Aachen, Germany

**Keywords:** animal model, antiseizure medications, *Drosophila melanogaster*, drug screening, epilepsy, seizure model

## Abstract

**Objective:**

Resistance to antiseizure medications (ASMs) is a major challenge in the treatment of patients with epilepsy. Despite numerous newly marketed ASMs, the proportion of drug-resistant people with epilepsy has not significantly decreased over the years. Therefore, novel and innovative seizure models for preclinical drug screening are highly desirable. Here, we explore the efficacy of a broad spectrum of ASMs in suppressing seizure activity in two established *Drosophila melanogaster* bang-sensitive mutants. These mutants respond with seizures to mechanical stimulation, providing a promising platform for screening novel ASMs.

**Methods:**

Seven frequently used ASMs (brivaracetam, cenobamate, lacosamide, lamotrigine, levetiracetam, phenytoin, and valproate) were administered to the bang-sensitive mutants *easily shocked* ^*2F*^ (*eas* ^*2F*^) and *paralytic* ^*bss1*^ (*para* ^*bss1*^). After 48 h of treatment, the flies were vortexed to induce mechanical stimulation. The seizure probability (i.e., ratio of seizing and non-seizing flies) as well as the seizure duration were analyzed.

**Results:**

In case of *eas* ^*2F*^ mutants, treatment with the sodium channel blockers phenytoin and lamotrigine resulted in a robust reduction of seizure probability, whereas flies treated with lacosamide showed a decrease in seizure duration. Treatment with valproate resulted in both a reduction in seizure probability and in seizure duration. In contrast, levetiracetam, brivaracetam and cenobamate had no effect on the bang-sensitive phenotype of *eas* ^*2F*^ flies. In case of *para* ^*bss1*^ flies, none of the tested medications significantly reduced seizure activity, supporting its role as a model of intractable epilepsy.

**Significance:**

Our results show that particularly sodium channel blockers as well as valproate are effective in suppressing seizure activity in the bang-sensitive mutant *eas* ^*2F*^. These findings demonstrate the usability of *Drosophila* for screening drugs with antiseizure properties. Due to fewer ethical concerns, the short life cycle, and low maintenance costs, *Drosophila* might provide an attractive and innovative high-throughput model for the discovery of novel antiseizure compounds.

## Introduction

Epilepsy is one of the most common chronic neurological disorders that affects more than 45 million people worldwide (Beghi et al., 2019). The main objective of epilepsy treatment is to achieve complete seizure control. However, about one-third of patients remain refractory to currently available antiseizure medications (ASMs) and experience ongoing seizures (Kwan and Brodie, 2000). Drug-resistant epilepsy is associated with an increased risk of psychosocial dysfunction, injuries, restricted quality of life, and premature death (Loscher et al., 2020). Thus, there is an unmet clinical need to develop more effective therapeutics, particularly with novel mechanisms of action.

The identification of potential novel therapeutics requires preclinical seizure models. A broad range of different animal models has been introduced over the years, such as the maximal electroshock seizure (MES) and the subcutaneous pentylenetetrazole (PTZ) seizure tests in rodents (Loscher, 2011; Loscher, 2017). However, despite the introduction of numerous new ASMs, the rate of drug-resistant people with epilepsy has not significantly declined (Chen et al., 2018). One possible explanation could be that current models might only identify substances with similar characteristics and therefore have no efficacy in case of drug-resistant epilepsies (Loscher, 2011). In addition, it must be considered that rodent models have some further limitations. For instance, the throughput of candidate compounds in drug screenings is considerably limited due to regulatory restrictions (e.g., by Institutional Animal Care and Use Committees), high costs as well as laborious experimental procedures. Additional model organisms enabling high-throughput screenings in a more cost-effective manner are highly desirable. Recent research has therefore focused on the development of alternative seizure models, including non-mammalian model organisms, such as roundworms, zebrafish, and fruit flies (Baraban, 2007; Cunliffe et al., 2015; Johan Arief et al., 2018; Rosch et al., 2019; Kasture et al., 2022; Fischer et al., 2023; Miguel Sanz et al., 2023; Shah et al., 2024).

The fruit fly *Drosophila melanogaster* has served as a model organism in biomedical research for more than a century (Bellen and Yamamoto, 2015) and has greatly advanced the understanding of many fundamental biological processes, such as genetics, inheritance, and development (Jennings, 2011; Yamaguchi and Yoshida, 2018). Among features that make *Drosophila* an attractive model organism are: fewer ethical restrictions (in line with the 3R principles - Replacement, Reduction, Refinement), a short generation time, the ease of maintenance, the cost-effectiveness, and the availability of a broad spectrum of sophisticated genetic tools (Jennings, 2011). The genome of the fly has been fully sequenced and encodes around 13,600 genes distributed across four pairs of chromosomes (Adams et al., 2000). Approximately 75% of all human disease-associated genes have a corresponding gene in *Drosophila*, making it a suitable model to study human disorders (Reiter et al., 2001). Despite obvious anatomical differences between fruit flies and humans, fundamental cellular and molecular processes show a high degree of similarity between these two species. The adult fly exhibits structures that are functionally analogous to the mammalian brain, heart, lung, kidney, gut, and reproductive system (Pandey and Nichols, 2011). The fly brain consists of some 100,000 neurons capable of mediating a wide range of complex behaviors, such as courtship, navigation, sleep, learning and memory (Pandey and Nichols, 2011). In addition, flies respond to various drugs that act within the central nervous system in a similar way to mammals (Pandey and Nichols, 2011), making it a suitable tool for drug screening (Desai et al., 2006; Chang et al., 2008).

The fruit fly has been used as a model to study epilepsy since the discovery of bang-sensitive mutants (for review see Fischer et al., 2023). These mutant flies characteristically respond to a mechanical shock, termed “bang”, with stereotypic seizure activity. This complex behavioral response can be divided into different phases, including an initial seizure, followed by temporary paralysis, and a recovery seizure (Song and Tanouye, 2008; Parker et al., 2011a; Fischer et al., 2023). A well-established example of a bang-sensitive mutant is *easily shocked* ^*2F*^ (*eas* ^*2F*^), carrying a variant in the gene encoding ethanolamine kinase, an enzyme involved in the synthesis of phosphatidylethanolamine. Presumably, mutations in this gene alter the phospholipid composition of membranes, which then leads to disturbances in neuronal excitability (Pavlidis et al., 1994). *Paralytic* ^*bss1*^ (*para* ^*bss1*^), another bang-sensitive fly line, harbors a gain-of-function mutation in the voltage-gated sodium channel gene *para* (Parker et al., 2011b), which is orthologous to the human *SCN1A* to *SCN5A* and *SCN7A* to *SCN11A* genes (Takai et al., 2020). This mutant is characterized by the most severe phenotype of all bang-sensitive mutants. Furthermore, it has been proposed as a model for intractable epilepsy because its phenotype was the most difficult to suppress by ASMs and seizure-suppressor mutations in previous analyses (Kroll et al., 2015).

In this study, we screened the capacity of seven frequently used ASMs to suppress seizure activity in two established *Drosophila* bang-sensitive mutants, namely *eas* ^*2F*^ and *para* ^*bss1*^. We show that several sodium channel blockers as well as valproate can suppress seizure activity in the *eas* ^*2F*^ mutant, whereas none of the tested drugs significantly influences the bang-sensitive phenotype of *para* ^*bss1*^ flies.

## Materials and methods

### Fly stocks and drug treatment

All flies were kept in plastic vials containing standard cornmeal food at 25°C. The *eas* ^*2F*^ flies were kindly donated by Richard Baines (University of Manchester, United Kingdom). The *para* ^*bss1*^ flies were a gift from Deepti Trivedi (National Centre for Biological Sciences, India). The Canton-S flies (#64349) were obtained from the Bloomington *Drosophila* Stock Center (IN, United States). The following drugs were used: brivaracetam (Tocris Bioscience), cenobamate (Alsachim), lacosamide (Sigma-Aldrich), lamotrigine (Selleck Chemicals), levetiracetam (Sigma-Aldrich), phenytoin (Sigma-Aldrich) and valproate (Sigma-Aldrich). Levetiracetam, brivaracetam and valproate were dissolved in water, whereas lacosamide, lamotrigine, phenytoin and cenobamate were dissolved in ethanol. In case of the *eas* ^*2F*^ mutant, three different drug concentrations were used, i.e., 0.03, 0.3 and 3 mM. These concentrations were selected based on a recommendation for pilot drug screenings in *Drosophila* (Pandey and Nichols, 2011). In case of *para* ^*bss1*^, we only used the highest concentration (3 mM). The method of drug preparation was adapted from Polet et al. (2024). 100 μL of the drug solution were equally distributed by pipet on top of the food and left to impregnate overnight. The concentrations noted in the graphs refer to the concentration of the solution added on top of the food (e.g., 100 μL of a 3 mM solution). Flies were kept on ASM containing food for 48 h. This period was chosen based on previous studies (e.g., Kasture et al., 2022). Solvent only treated flies served as control.

### Vortex assay

Adult male flies were collected using CO_2_ 1–3 days after eclosion and transferred to a food vial prepared with the respective drug (max. 10 flies per vial). After 48 h, the flies were transferred to an empty vial (max. 5 flies per vial) and allowed to recover from anesthesia for 1–2 h. For seizure induction, the vials were vortexed using a Vortex-Genie 2 (Scientific Industries) at maximum speed for 10 s as described previously (Kuebler and Tanouye, 2000; Mituzaite et al., 2021). The seizure activity was recorded using a video camera. The ratio of seizing and non-seizing flies (termed seizure probability) as well as the seizure duration were measured. The seizure duration was defined as the time from the end of the vortex stimulation until the fly regained posture and mobility.

### Statistical analysis

To test whether the rate of seizing flies differed between the testing conditions, we performed a Fisher’s exact test. For seizure duration, statistical outliers were excluded using the ROUTE method, and normal distribution was confirmed by Shapiro-Wilk test. The seizure duration was analyzed by a one-way ANOVA followed by Tukey’s *post hoc* t-tests. Statistical significance was set at *p* < 0.05 with *p < 0.05, **p < 0.01, ***p < 0.001. All error bars shown represent the standard deviation (SD).

## Results

### Effects of different solvents on the seizure activity in *eas^2F^* flies

To validate the utility of *Drosophila* as a screening tool for antiseizure compounds, we fed two established bang-sensitive mutants with a broad spectrum of frequently used ASMs for 48 h. The tested drugs comprised (i) sodium channel blockers (phenytoin, lacosamide, lamotrigine), (ii) synaptic vesicle glycoprotein 2 A (SV2A) modulators (levetiracetam, brivaracetam) and (iii) drugs with multiple mechanisms of action (valproate, cenobamate). Drug consumption was verified using food coloring (Supplementary Figure S1). For the initial screening, we chose the *eas* ^*2F*^ mutant because it features a relatively short seizure duration compared to other bang-sensitive mutants and is thus a more suitable model for large-scale screens. The seizure activity was induced by vortex stimulation (Kuebler and Tanouye, 2000; Mituzaite et al., 2021) and the seizure probability and seizure duration were measured.

**FIGURE 1:**
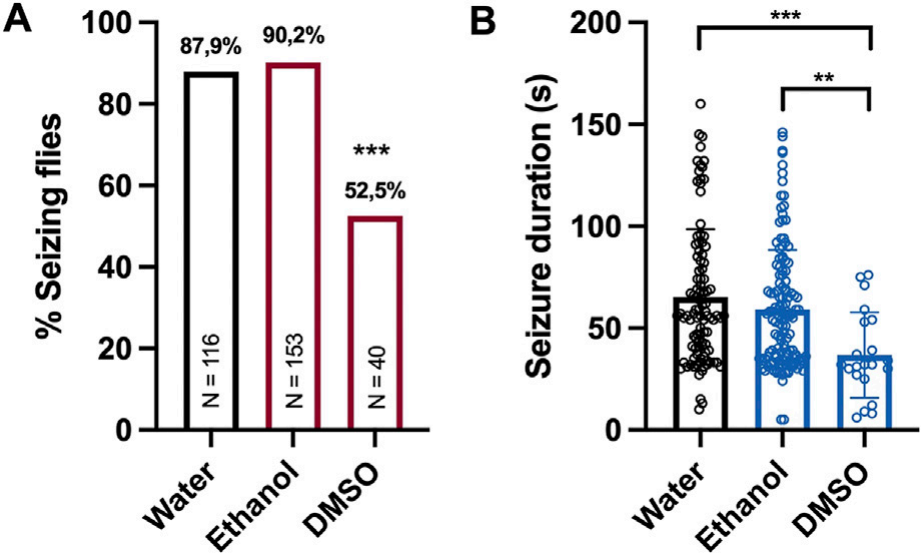
Seizure activity of *eas* ^*2F*^ flies treated with different solvents **(A)** Percentage of seizing *eas* ^*2F*^ flies after 10 s of vortex stimulation. Before testing, the flies were fed with 100 μL of water, ethanol or DMSO for 48 h. The data for flies treated with water or ethanol were derived from experiments shown in Figure 2. For statistical analysis, we performed a Fisher’s exact test (***p < 0.001, compared to water and ethanol). N indicates the total number of flies tested per condition. **(B)** Seizure duration in seconds of the same *eas* ^*2F*^ flies. The data was analyzed by a one-way ANOVA followed by Tukey’s *post hoc* t-tests (**p < 0.01, ***p < 0.001). Error bars indicate SD.

To analyze possible confounding effects of the solvent on the seizure activity, we compared the seizure activity of *eas* ^*2F*^ flies treated with either water, ethanol, or dimethyl sulfoxide (DMSO) alone. Treatment with DMSO lead to a significant decrease in seizure probability and seizure duration. In contrast, no differences were observed between flies treated with water or ethanol (Figures 1A, B). Therefore, we used ethanol as a solvent for hydrophobic agents instead of DMSO in our screening experiments.

### Influence of ASM treatment on seizure activity in *eas^2F^* flies

**FIGURE 2:**
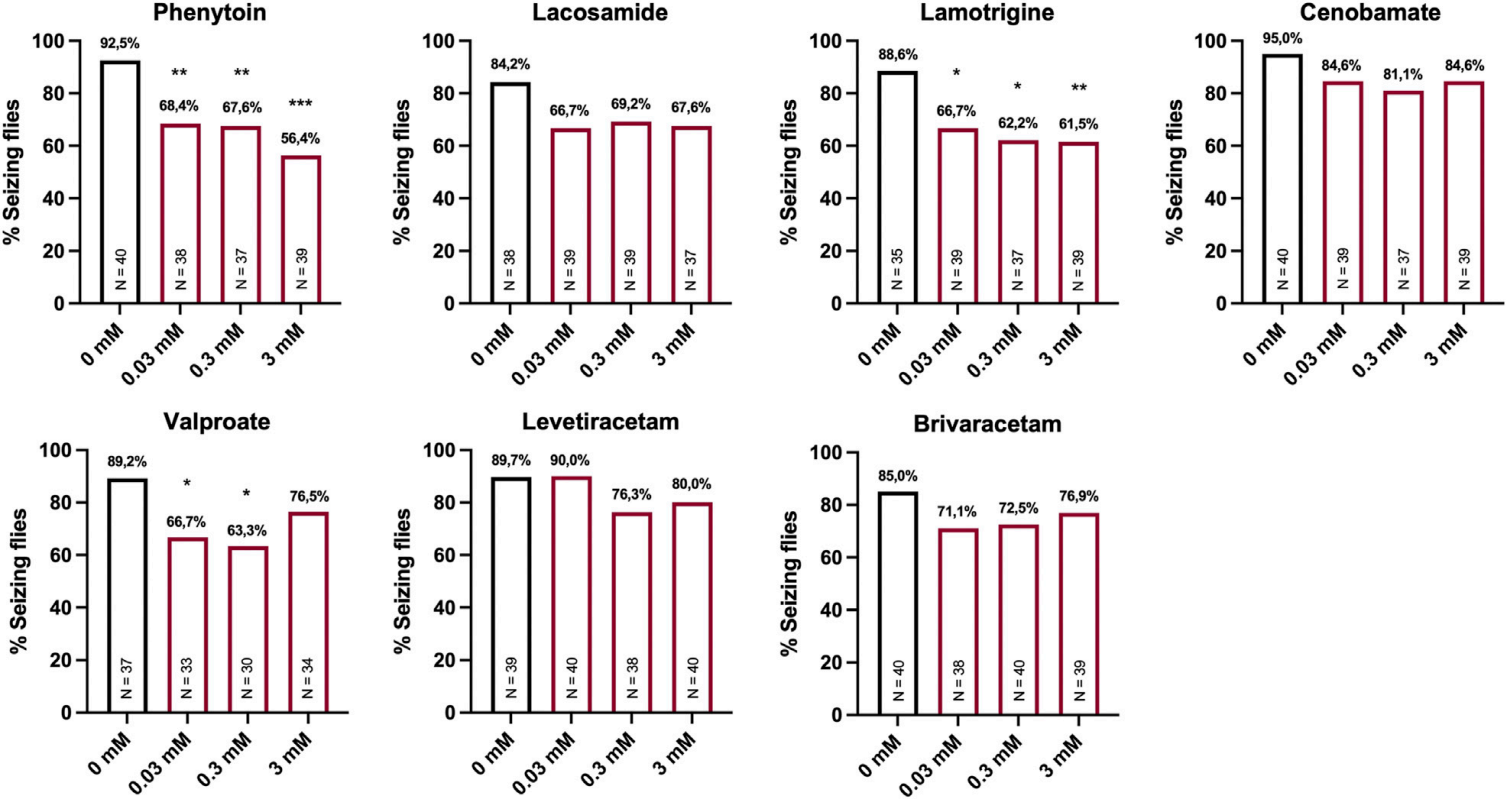
Seizure probability of *eas* ^*2F*^ flies treated with seven different ASMs. Percentage of seizing flies after 10 s of vortex stimulation. Before testing, the flies were treated with three different concentrations (0.03, 0.3, 3 mM) of seven different ASMs (phenytoin, lacosamide, lamotrigine, cenobamate, valproate, levetiracetam, brivaracetam) for 48 h. For statistical analysis, we performed a Fisher’s exact test (*p < 0.05, **p < 0.01, ***p < 0.001, compared to 0 mM). N indicates the total number of tested flies per condition.

**FIGURE 3:**
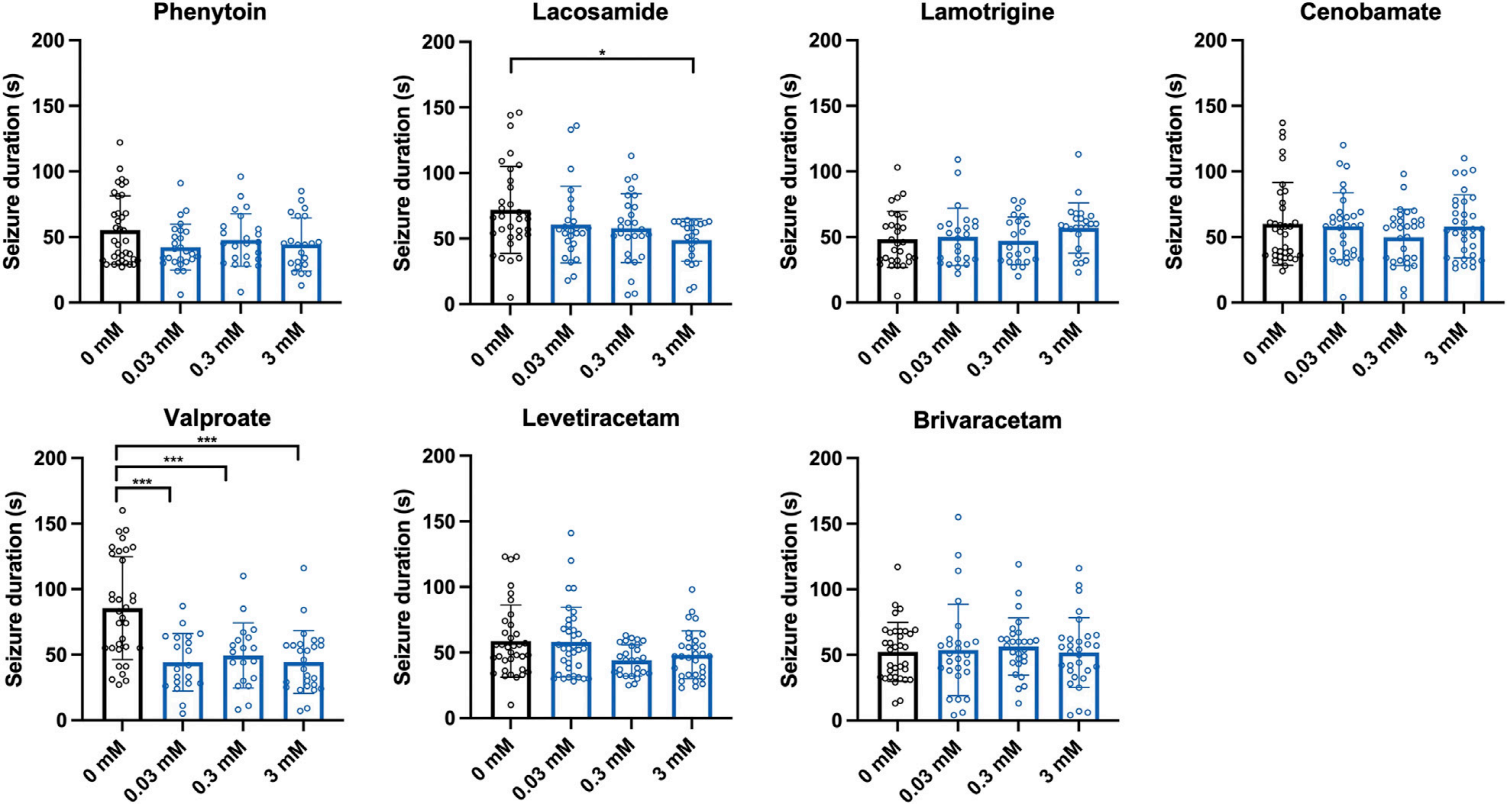
Seizure duration of *eas* ^*2F*^ flies treated with seven different ASMs. Seizure duration in seconds of flies after 10 s of vortex stimulation. Before testing, the flies were treated with three different concentrations (0.03, 0.3, 3 mM) of seven different ASMs (phenytoin, lacosamide, lamotrigine, cenobamate, valproate, levetiracetam, brivaracetam) for 48 h. For statistical analysis, we performed a one-way ANOVA followed by Tukey’s *post hoc* t-tests (*p < 0.05, ***p < 0.001, compared to 0 mM). Error bars indicate SD.

In our screening paradigm, we tested three different concentrations (0.03, 0.3, and 3 mM) of each ASM, based on a recommendation for pilot drugs screenings in *Drosophila* (Pandey and Nichols, 2011). Treatment with the sodium channel blockers phenytoin and lamotrigine resulted in a dose-dependent reduction of seizure probability, whereas no significant effect was observed for the sodium channel blocker lacosamide (Figure 2). In addition to phenytoin and lamotrigine, a robust decrease in seizure probability was observed in flies treated with valproate (Figure 2). Interestingly, this effect was only significant for 0.03 and 0.3 mM but not for 3 mM. In contrast, no effects on the seizure probability were observed for the SV2A modulators levetiracetam and brivaracetam as well as for cenobamate (Figure 2). Next, we investigated the effects of the same ASMs on the seizure duration of *eas* ^*2F*^ flies. Here, flies treated with lacosamide showed a significantly shorter seizure duration than the control flies, but only at the highest concentration (3 mM) (Figure 3). In addition, flies treated with valproate also showed a significantly reduced seizure duration, whereas no changes were observed for phenytoin, lacosamide, levetiracetam, brivaracetam, and cenobamate (Figure 3).

### Confirmation of *para^bss1^* as a model of refractory epilepsy

**FIGURE 4:**
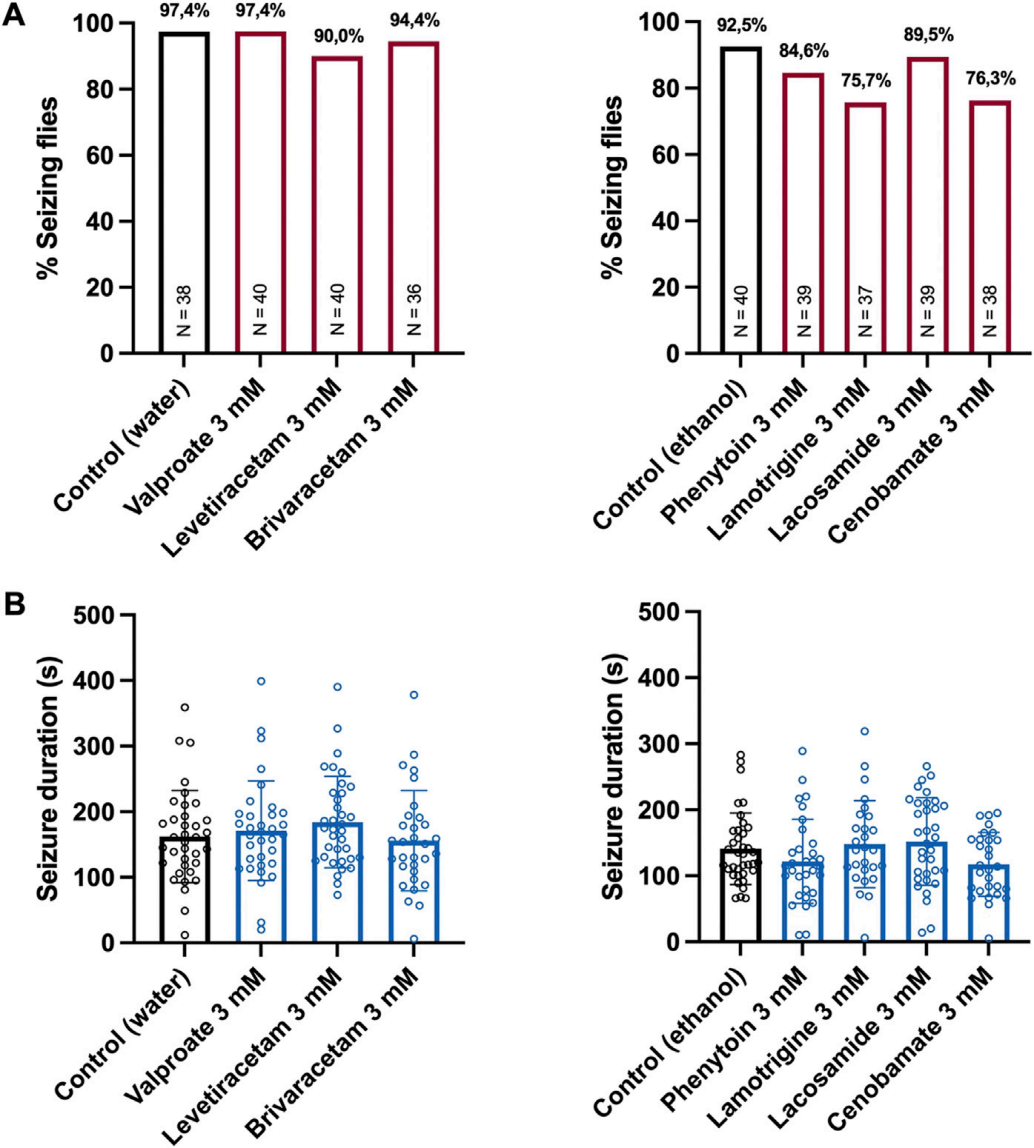
Seizure activity of *para* ^*bss1*^ flies treated with seven different ASMs. **(A)** Percentage of seizing flies after 10 s of vortex stimulation. Before testing, the flies were treated with 3 mM of seven different ASMs (phenytoin, lacosamide, lamotrigine, cenobamate, valproate, levetiracetam, brivaracetam) for 48 h. For statistical analysis, we performed a Fisher’s exact test (no significance, compared to control). N indicates the total number of tested flies per condition. **(B)** Seizure duration in seconds of the same flies described above. For statistical analysis, we performed a one-way ANOVA followed by Tukey’s *post hoc* t-tests (no significance, compared to control). Error bars indicate SD.

To investigate whether the observed effects on the seizure activity are specific for the *eas* ^*2F*^ flies, we additionally tested the same ASMs in *para* ^*bss1*^, another bang-sensitive mutant and model of intractable epilepsy. Here, we only tested the highest concentration (3 mM) of each ASM. None of our ASMs showed a significant effect on the seizure probability of these flies (Figure 4A). However, we noted a tendency for some sodium channel inhibitors (lamotrigine and cenobamate) to reduce the rate of seizing flies, which seems not surprising as *para* ^*bss1*^ is known to carry a gain-of-function mutation in the voltage-gated sodium channel. In addition, none of our ASMs had a significant effect on the seizure duration of *para* ^*bss1*^ flies (Figure 4B). Importantly, these results confirm *para* ^*bss1*^ as a model of refractory epilepsy.

## Discussion

To demonstrate the utility of the fruit fly *Drosophila* as a screening tool for novel antiseizure compounds, we tested the capacity of seven frequently used ASMs to suppress seizure activity in two established bang-sensitive mutants, *eas* ^*2F*^ and *para* ^*bss1*^. To avoid possible confounding effects caused by the solvents in which the ASMs are dissolved, we also investigated the effects of water, ethanol, and DMSO on the seizure activity of the *eas* ^*2F*^ mutant. Surprisingly, we found that treatment with DMSO alone caused a strong decrease in seizure probability and seizure duration, whereas no significant differences were found between flies treated with water or ethanol. The exact mechanisms of how DMSO suppressed seizure activity in this model are not clear. However, DMSO is known to block activation of sodium channels (Larsen et al., 1996) and to decrease GABA-, NMDA- and AMPA-induced ion currents (Nakahiro et al., 1992; Lu and Mattson, 2001). These effects might alter the balance between inhibition and excitation and thus affect neuronal excitability. Our findings are also consistent with a recent study demonstrating a short-lasting decrease in epileptiform activity in a mouse model of chronic temporal lobe epilepsy after injection of 100% DMSO (Widmann et al., 2023). Therefore, the use of DMSO as a solvent should be evaluated cautiously when studying seizure activity in *Drosophila* bang-sensitive mutants. A potential antiseizure effect of DMSO should be monitored closely by control experiments.

In case of *eas* ^*2F*^ flies, we found that treatment with the sodium channel blockers phenytoin and lamotrigine caused a robust reduction in seizure probability, whereas treatment with lacosamide resulted in a decrease in seizure duration. Interestingly, cenobamate, which is also classified as a sodium channel blocker, had no significant effect on the seizure activity of *eas* ^*2F*^ flies. These divergent effects among sodium channel blockers might be explained by subtle differences in their mechanism of action. For instance, phenytoin and lamotrigine exert their antiseizure effects predominantly by enhancing fast inactivation of sodium channels, whereas the sodium channel blocker lacosamide primarily increases slow inactivation (Rogawski et al., 2015). In contrast, cenobamate has a pronounced effect on persistent rather than transient sodium currents. In addition, it is also an allosteric modulator of GABA_A_ receptors (Roberti et al., 2021). Apart from phenytoin, lamotrigine and lacosamide, also valproate showed a significant effect on the seizure activity of *eas* ^*2F*^ flies. Notably, valproate caused both a reduction in seizure probability and in seizure duration. This pronounced effect might be due to the combination of different mechanisms by which valproate exerts its antiseizure effects, including inhibition of voltage-gated sodium and T-type calcium channels as well as by increasing the concentration of GABA (Broicher et al., 2007; Loscher and Klein, 2021). Of note, the effect on seizure probability was not significant at the highest concentration, which might be explained by toxic effects that have been reported at higher doses (Polet et al., 2024). No significant effects on the seizure activity were found for the SV2A modulators levetiracetam and brivaracetam. There are several possible reasons for this observation. For instance, the binding to the *Drosophila* orthologue of SV2A, CG3168, might be insufficient or the *Drosophila* orthologue is functionally not amply identical to the human SV2A. Interestingly, levetiracetam was also devoid of any antiseizure activity in two well-established screening models for ASMs - the MES and PTZ seizure tests - highlighting its unique profile among current ASMs (Klitgaard et al., 1998). In addition to *eas* ^*2F*^, we also tested the same ASMs in another bang-sensitive mutant, i.e., *para* ^*bss1*^. This mutant carries a gain-of-function mutation in the *Drosophila* voltage-gated sodium channel (Parker et al., 2011b) and is characterized by the most severe phenotype of all bang-sensitive mutants. Moreover, its phenotype is the most difficult to suppress by ASMs or seizure suppressor mutations. Hence, it has been proposed as a model of intractable epilepsy (Kroll et al., 2015). Concordantly, none of our tested drugs had a significant effect on the seizure probability or seizure duration of this mutant, supporting its role as a model of refractory epilepsy. However, it should be noted that we only tested the highest concentration of each ASM in the *para* ^*bss1*^ mutant. Hence, we cannot rule out a potential effect at lower doses, e.g., of valproate as seen in the *eas* ^*2F*^ mutant. Importantly, variability in drug response is also commonly observed in people with epilepsy, a phenomenon attributed to genetic differences, the heterogeneous nature of epilepsy syndromes, and individual variations in drug metabolism (Heavin et al., 2019; Wolking et al., 2020a; Wolking et al., 2020b; Wolking et al., 2021). As demonstrated in this study, the use of *Drosophila* mutants effectively models this variability. Investigating why certain drugs are not effective in one mutant but succeed in another could reveal new targets for ASMs or novel mechanisms of drug resistance, which could be pivotal for overcoming treatment-resistant forms of epilepsy in humans.

*Drosophila* offers several advantages as a model for drug screening, including fewer ethical concerns, low maintenance costs, and high progeny numbers for robust statistical analysis. Furthermore, screening novel drug candidates in an *in vivo* model, such as *Drosophila*, enables the identification of high-quality hits that already exhibit key features such as oral availability, metabolic stability, and low toxicity (Pandey and Nichols, 2011). These characteristics cannot yet be adequately mimicked in cell culture experiments or biochemical assays (Pandey and Nichols, 2011). Another advantage of *Drosophila* is the ease of generating transgenic flies expressing human disease-associated variants (Link and Bellen, 2020), offering a promising approach to test personalized therapy strategies. Indeed, there are already several studies exploring disease-specific therapies in *Drosophila*. For instance, a recent study investigated the effectiveness of several ASMs in a *Drosophila* model of *KCNT1*-epilepsy (Hussain et al., 2024). The authors found that cannabidiol decreased the seizure phenotype of flies expressing the human *KCNT1* gene in GABAergic neurons with three different patient mutations. Furthermore, they showed that vigabatrin significantly reduced the seizure activity in two of the tested mutants, but increased seizure activity in the third one. Another study explored the efficacy of several ASMs in a *Drosophila* model of North Sea progressive myoclonus epilepsy (Polet et al., 2024). The authors showed that treatment with sodium barbital, clonazepam, ganaxolone and ethosuximide resulted in a significant reduction of heat-induced seizures in flies with glial knockdown of *Membrin*, the orthologue of human *GOSR2*. Another study found that the serotonin precursor 5-hydroxytryptophan (5-HTP) suppressed heat-induced seizures in a *Drosophila* model of Dravet Syndrome but increased seizure-activity in a model of genetic epilepsy with febrile seizures plus (GEFS+) (Schutte et al., 2014). Importantly, these studies further support *Drosophila* as a model for drug screening.

However, some limitations should be considered when using whole animals in large-scale screens. For instance, the actual physiological concentrations of candidate compounds within the model organism can hardly be predicted. Therefore, it is hard to interpret whether negative findings result from poor absorption, distribution issues, or an actual absence of biological activity (Giacomotto and Segalat, 2010). At least in our case, food coloring was used to verify consumption of the drugs. However, it is noteworthy that it is still hardly possible to determine the exact amount of the ingested drug. Hence, we cannot rule out subtle differences in drug intake. Furthermore, the duration of treatment that is required to achieve a positive effect is difficult to estimate. Thus, we cannot rule out that a longer treatment would have resulted in a stronger antiseizure effect in our experiments. However, the main purpose of using small animals in large-scale screens is to streamline the pool of candidate compounds to those of higher quality, which ultimately promises both time and cost efficiency (Giacomotto and Segalat, 2010).

## Conclusion

In the present study, we screened the capacity of a broad range of ASMs to suppress seizures in two established *Drosophila* bang-sensitive mutants. We found a distinct, ASM-dependent pattern of seizure response for sodium channel blockers and valproate in *eas* ^*2F*^ flies and broad drug-resistance in *para* ^*bss1*^ flies. Our results imply that the fruit fly might be a suitable model for large-scale screening of anti-seizure agents, promising a higher pace and throughput, and bearing the potential to advance the discovery of novel antiseizure compounds. Finally, we want to emphasize that the fruit fly is not meant to replace rodent and other animal models in drug discovery but complement current drug discovery pipelines as a high-throughput instrument.

## Data Availability

The raw data supporting the conclusions of this article will be made available by the authors, without undue reservation.

## Supplementary material

SUPPLEMENTARY FIGURE S1 Verification of drug intake. The following drugs were diluted with food coloring (brilliant blue FCF; E 133) to a final concentration of 3 mM: brivaracetam (BRV), cenobamate (CBM), lacosamide (LCM), lamotrigine (LTG), levetiracetam (LEV), phenytoin (PHT), valproate (VPA). 100 µL of the drug/food coloring solution were equally distributed by pipet on top of the food and left to impregnate overnight. Adult male flies (Canton-S) were collected using CO_2_ 1–3 days after eclosion and transferred to a food vial prepared with the respective drug/food coloring solution (10 flies per vial). After 48 h, the abdomen of each fly was visually inspected under a microscope. All flies exhibited a blue colored abdomen, confirming ingestion of each ASM. Untreated flies (without food coloring) served as control.
