# Supplementary figures and images for: The fruit fly Drosophila melanogaster as a screening model for antiseizure medications
Source: Front Pharmacol. 2024 Dec 10;15:1489888. doi: 10.3389/fphar.2024.1489888 (PMC11666373; doi:10.3389/fphar.2024.1489888)

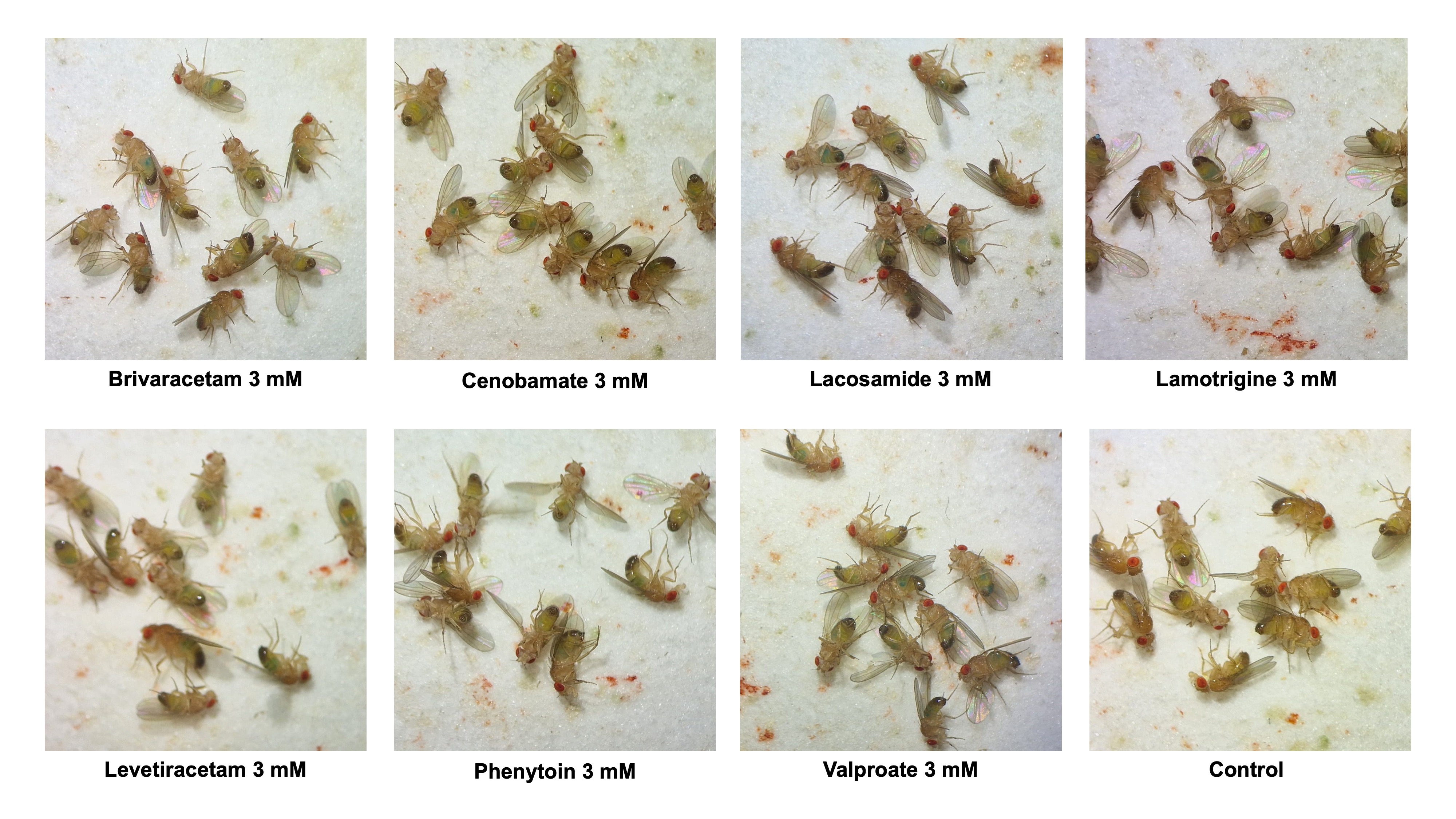

Supplement: Supplementary file 1 [file Image1.JPEG]
